# Thymus-derived glucocorticoids are insufficient for normal thymus homeostasis in the adult mouse

**DOI:** 10.1186/1471-2172-5-24

**Published:** 2004-11-02

**Authors:** Stephen B Pruett, Eric L Padgett

**Affiliations:** 1 Department of Cellular Biology and Anatomy, Louisiana State University Health Sciences Center, 1501 Kings Highway, Shreveport, LA, USA; 2 Wil Research Labs, 1407 George Road, Ashland, OH, USA

## Abstract

**Background:**

It is unclear if thymus-derived glucocorticoids reach sufficient local concentrations to support normal thymus homeostasis, or if adrenal-derived glucocorticoids from the circulation are required. Modern approaches to this issue (transgenic mice that under or over express glucocorticoid receptor in the thymus) have yielded irreconcilably contradictory results, suggesting fundamental problems with one or more the transgenic mouse strains used. In the present study, a more direct approach was used, in which mice were adrenalectomized with or without restoration of circulating corticosterone using timed release pellets. Reversal of the increased number of thymocytes caused by adrenalectomy following restoration of physiological corticosterone concentrations would indicate that corticosterone is the major adrenal product involved in thymic homeostasis.

**Results:**

A clear relationship was observed between systemic corticosterone concentration, thymus cell number, and percentage of apoptotic thymocytes. Physiological concentrations of corticosterone in adrenalectomized mice restored thymus cell number to normal values and revealed differential sensitivity of thymocyte subpopulations to physiological and stress-inducible corticosterone concentrations.

**Conclusion:**

This indicates that thymus-derived glucocorticoids are not sufficient to maintain normal levels of death by neglect in the thymus, but that apoptosis and possibly other mechanisms induced by physiological, non stress-induced levels of adrenal-derived corticosterone are responsible for keeping the total number of thymocytes within the normal range.

## Background

Although it is clear that elevated concentrations of endogenous glucocorticoids can cause apoptosis in the thymus [1-3], the role of normal concentrations of glucocorticoids in thymic homeostasis remains controversial [4-7]. Results reported by Ashwell and colleagues suggest glucocorticoids are essential at very low concentrations for early development and survival of thymocytes and that glucocorticoids can alter the sensitivity of more mature thymocytes to positive selection, thereby influencing the T cell receptor repertoire [5]. In addition, there is convincing evidence that corticosterone is produced in the thymus and that it acts locally to affect thymocyte development [8,9]. Therefore, it was surprising when normal cellular development (including repertoire) was observed until the time of birth in glucocorticoid receptor knockout mice [10]. This raised serious questions about the necessity of glucocorticoids as a required or permissive agent in thymic development.

It has also been suggested that glucocorticoids play a role in homeostasis in the adult thymus by inducing death by neglect of thymocytes that are neither positively nor negatively selected. This idea has been based on the observation that the predominant cell type subjected to death by neglect, CD4^+^CD8^+ ^non-mature thymocytes, is most susceptible to elevated concentrations of glucocorticoids [11]. Recent results indicate that overexpression of glucocorticoid receptors (GR) in developing and mature T cells leads to decreased cell number in the thymus and a decreased number of T cells in the periphery in adult mice. In addition, decreased expression of GR is associated with increased cell number in the thymus [4]. However, results obtained with knockout or transgenic mice have been contradictory [4,7,12-14]. For example, one group using transgenic mice that express anti-sense GR mRNA in the thymus found increased thymus cellularity [4], whereas another group using a cre-lox conditional knockout system to eliminate glucocorticoid receptor in cells that express CD4 (including double positive cells) reported no increase in cellularity [14]. Both groups verified that the expected decrease in sensitivity to high concentrations of glucocorticoids occurred in the transgenic mice. Until the basis for such differences can be determined, it seems reasonable to use an alternate approach that does not alter the glucocorticoid receptor (except by natural mechanisms relating to glucocorticoid concentration). In addition, transgenic approaches cannot provide the concentration-response information for corticosterone that would be needed to distinguish normal physiological effects and stress-related effects.

A small number of studies have been reported in which systemic glucocorticoid concentrations were reduced by adrenalectomy, leading to increased numbers of cells in the thymus [15-17]. However, this observation has not been universal, with one report indicating no increase in thymus cellularity in adrenalectomized mice [18]. Thus, confirmation of an adrenalectomy-induced increase in thymus cellularity would be useful. Even if confirmation is obtained, it would still be possible that an adrenal product other than corticosterone was responsible for increased cell number in the thymus. However, if corticosterone was the major regulator of thymus homeostasis, restoring corticosterone to physiological levels in adrenalectomized mice should return thymus cell number and subpopulation ratios to normal values. Therefore, this approach was used in the present study to determine the role of systemic corticosterone in thymus homeostasis. The study was designed so the results would also indicate whether thymus-derived corticosterone is sufficient to permit normal maintenance of number of cells in each major subpopulation in the thymus.

If physiological (adrenal-derived) concentrations of corticosterone are important in the induction of death by neglect of thymocytes, it would seem likely that any increase in cell number in the thymus of adrenalectomized mice would be explained mostly by an increase of CD4^+^CD8^+ ^cells, which are the predominant cell type subjected to death by neglect [11]. In addition, it has also been proposed that physiological concentrations of corticosterone increase the sensitivity of thymocytes to negative selection. Preventing this would presumably cause fewer single positive thymocytes to die, thus increasing the percentages of these cells in the thymus. It might also be expected that immature single positive thymocytes (CD3 low, CD4^-^CD8^+^) would be increased, as these cells have been reported to be particularly sensitive to glucocorticoids [19]. If physiological (non-stress) concentrations of corticosterone contribute to the induction of death by neglect or negative selection, it would be expected that sub-physiological concentrations of corticosterone would decrease apoptosis in the thymus. Failure to observe these changes in mice with sub-physiological concentrations of corticosterone would suggest either that thymus-derived corticosterone [8] is sufficient to compensate for loss of systemic (adrenal-derived) corticosterone or that corticosterone is not directly involved in these processes under physiological, non-stress conditions. The studies described here were designed to directly test these predictions and thus to indirectly evaluate the role of endogenous glucocorticoids in death by neglect in the thymus. In addition, this study was designed to distinguish the relative contributions of systemic (mostly adrenal-derived) glucocorticoids and those produced in the thymus [8]. The use of a dose-response approach permitted identification of the point on the corticosterone concentration vs. thymocyte subpopulation plot that corresponds to a physiological corticosterone concentration, and it permitted identification of a distinction between the effects of sub-physiological concentrations of corticosterone and stress-inducible concentrations.

## Results and discussion

### Adrenalectomy increases cell number and alters subpopulation percentages in the thymus, and this effect is inhibited 24 hr after restoration of corticosterone

**Figure 1:**
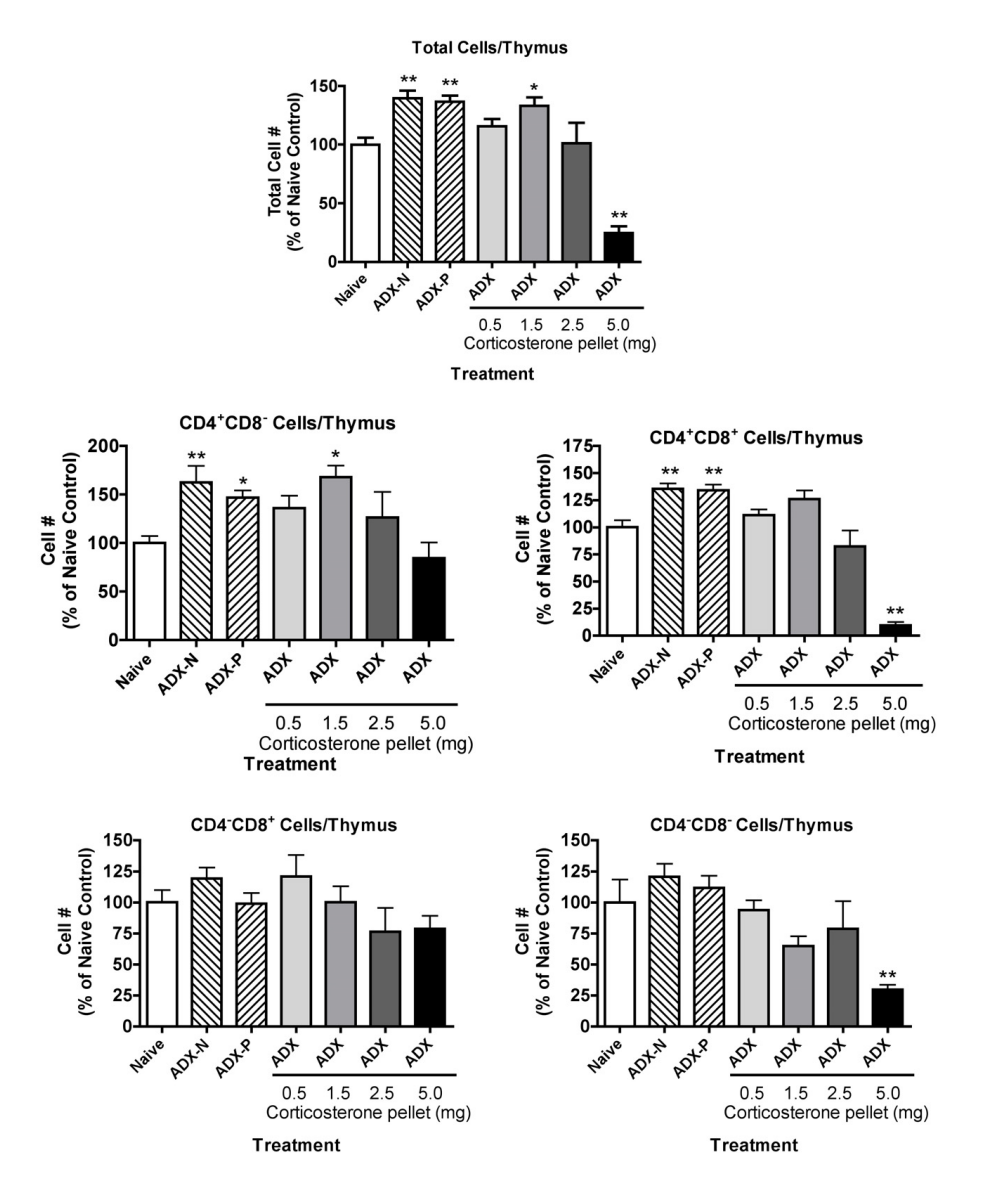
**Effect of adrenalectomy and restoration of various concentrations of corticosterone on the number of nucleated cells in the thymus 24 hours after implantation of corticosterone pellets **Treatment groups were: Naive, untreated; ADX-N, adrenalectomized naive; ADX-P, adrenalectomized with placebo pellet implanted; other groups, adrenalectomized with corticosterone pellets of the indicated size implanted. The thymus was evaluated 24 hours after pellet implantation (~3–4 weeks after ADX). Group size was 5–12. Values shown are mean ± S.E. obtained by normalizing groups to the mean value for the Naive group (defined as 100%). Results shown are pooled from two independent experiments, and groups significantly different from the naive group (by ANOVA followed by Dunnett's test) are shown by * (*p *< 0.05) or ** (*p *< 0.01).

The results shown in Figure 1 demonstrate that adrenalectomy significantly increases the total number of cells in the thymus as well as the number of CD4^+^CD8^- ^and CD4^+^CD8^+ ^cells. The number of CD4^-^CD8^+ ^and CD4^-^CD8^- ^cells was also greater in adrenalectomized mice than in the naive control group, but the difference was not significant. This indicates that the number of CD4^+^CD8^- ^and CD4^+^CD8^+ ^cells is diminished to a greater extent than CD4^-^CD8^+ ^and CD4^-^CD8^- ^cells by normal, physiological concentrations of corticosterone. Results for adrenalectomized mice were comparable whether a placebo pellet was implanted or not, indicating that non-adrenal-derived stress mediators induced by pellet implantation did not affect cell number. Timed release corticosterone pellets were used to restore corticosterone in adrenalectomized mice, and 0.5 and 1.5 mg pellets had only minimal effects on any subpopulation 24 hours after implantation of pellets (Figure 1). Pellets containing 2.5 mg of corticosterone returned the number of CD4^+^CD8^- ^and CD4^+^CD8^+ ^cells to near normal values. A pellet containing 5.0 mg of corticosterone significantly decreased the number of CD4^-^CD8^- ^and CD4^+^CD8^+ ^cells as compared to the naive control group, suggesting that CD4^-^CD8^- ^and CD4^+^CD8^+ ^are more sensitive than CD4^+^CD8^- ^and CD4^-^CD8^+ ^cells to stress-inducible corticosterone concentrations. The greater sensitivity of CD4^+^CD8^+ ^cells as compared to the other subpopulations at high corticosterone concentrations has prompted an assumption that these cells are more sensitive to physiological (unstressed) concentrations of corticosterone. However, this was not supported by the results presented here. The results in Figure 1 do indicate that CD4^+^CD8^+ ^cells increase in number to a greater extent than CD4^-^CD8^+ ^or CD4^-^CD8^- ^cells in ADX mice, but CD4^+^CD8^- ^cells increase in number proportionally more than all these other subpopulations in mice with sub-physiological concentrations of corticosterone (ADX groups).

**Figure 2:**
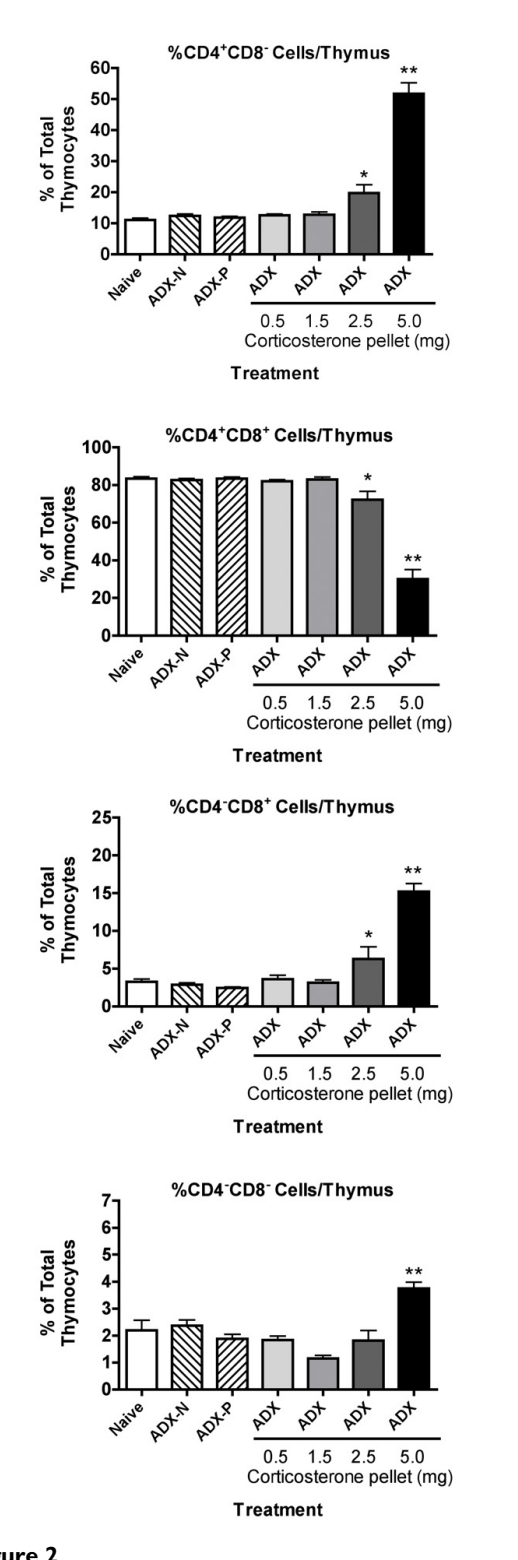
**Effect of adrenalectomy and restoration of various concentrations of corticosterone on the percentage of cells in the 4 major subpopulations in the thymus 24 hours after implantation of corticosterone pellets **Data from the experiments noted in Figure 1 were analyzed for changes in the percentage of each cell type as compared to the total number of nucleated cells per thymus. The values did not differ significantly between experiments for any subpopulation, so the data were pooled (without normalizing). Groups significantly different from the naive group (by ANOVA followed by Dunnett's test) are shown by * (*p *< 0.05) or ** (*p *< 0.01).

The changes in subpopulation percentages (as opposed to cell numbers, which are shown in Figure 1) in the thymus 24 hours after implantation of pellets are shown in Figure 2. These results confirm the greater sensitivity of CD4^+^CD8^+ ^cells than the other subpopulations to stress-inducible concentrations of corticosterone (in mice with a 5 mg pellet). The greater sensitivity of CD4^-^CD8^- ^cells than CD4^-^CD8^+ ^and CD4^+^CD8^- ^cells noted in Figure 1, was also evident in Figure 2 in terms of a lesser increase in percentage of the former cell type as compared to the latter cell types in mice treated with a 5 mg pellet. However, it should be emphasized that all subpopulations decreased in absolute number in these mice, so these percentage values do not reflect increases in the number of cells in these subpopulations but increases relative to CD4^+^CD8^+ ^cells, which are the most abundant and were diminished to the greatest extent. No significant change in subpopulation percentages was caused by adrenalectomy with or without a placebo pellet. However, it should be noted that the results shown in Figure 1 indicate that the number of CD4^+^CD8^+ ^and CD4^+^CD8^- ^cells increased to a greater extent than the number of CD4^-^CD8^- ^or CD4^-^CD8^+ ^cells. This did not result in a substantial change in percentages (Figure 2), because the cells that increased the most (CD4^+^CD8^+ ^cells) account for over 90% of the total thymocyte population, and changes in this subpopulation were reflected in the denominator (total cell number) of the equation that determines the percentage of cells in each population.

### Adrenalectomy increases cell number and alters subpopulation percentages in the thymus, and this effect is inhibited 72 hr after restoration of corticosterone

**Figure 3:**
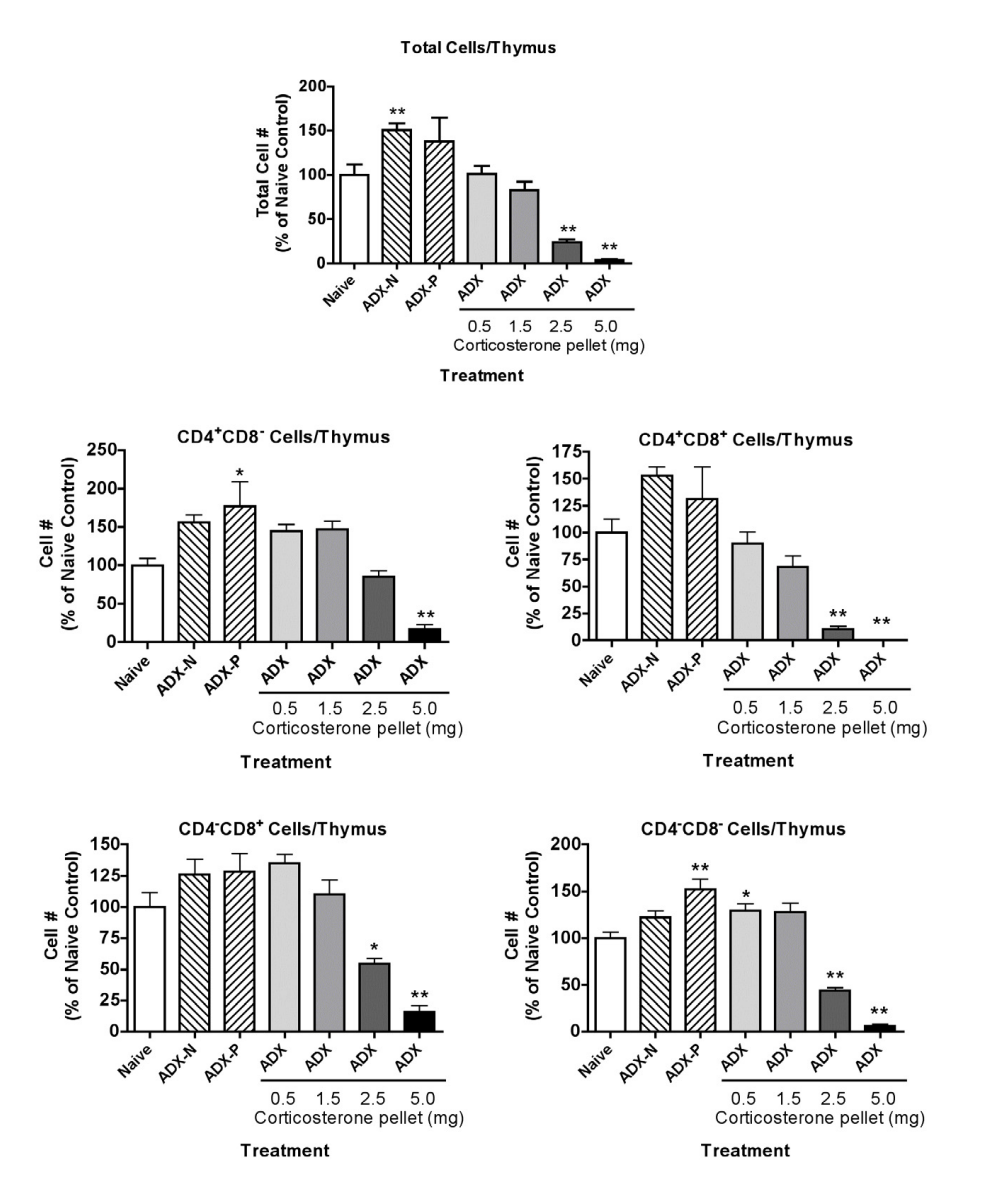
**Effect of adrenalectomy and restoration of various concentrations of corticosterone on the number of nucleated cells in the thymus 72 hours after implantation of corticosterone pellets **Treatment groups were: Naive, untreated; ADX-N, adrenalectomized naive; ADX-P, adrenalectomized with placebo pellet implanted; other groups, adrenalectomized with corticosterone pellets of the indicated size implanted. The thymus was evaluated 72 hours after pellet implantation (~3–4 weeks after ADX). Group size was 5–12. Values shown are mean ± S.E. obtained by normalizing groups to the mean value for the Naive group (defined as 100%). Results shown are pooled from two independent experiments, and groups significantly different from the naive group (by ANOVA followed by Dunnett's test) are shown by * (*p *< 0.05) or ** (*p *< 0.01).

**Figure 4:**
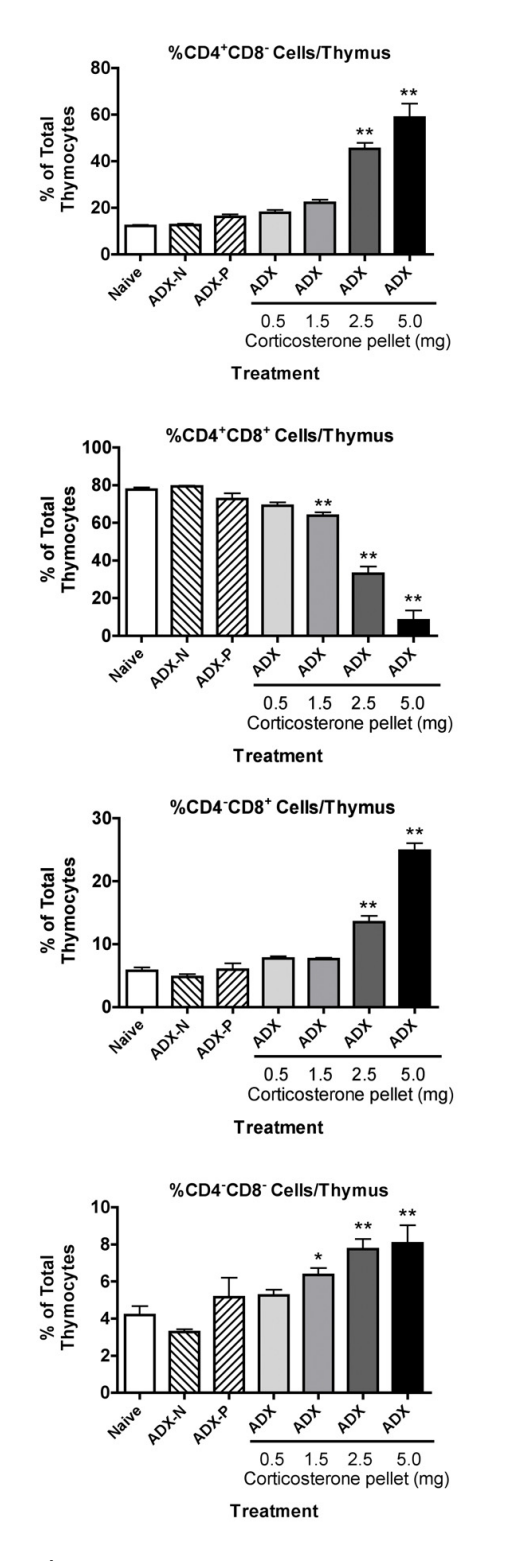
**Effect of adrenalectomy and restoration of various concentrations of corticosterone on the percentage of cells in the 4 major subpopulations in the thymus 72 hours after implantation of corticosterone pellets **Data from the experiments noted in Figure 3 were analyzed for changes in the percentage of each cell type as compared to the total number of nucleated cells per thymus. Groups significantly different from the naive group (by ANOVA followed by Dunnett's test) are shown by * (*p *< 0.05) or ** (*p *< 0.01).

Changes in the thymus were first evaluated 24 hours after implantation of pellets to allow determination of the role of apoptosis in loss of cells at a time point at which cell numbers were still decreasing. To determine if greater or different effects were evident after a longer period of corticosterone exposure, thymuses were evaluated 72 hours after implantation of pellets. As shown in Figure 3, the effects on cell number and on the number of cells in most subpopulations were greater than observed after 24 hours (Figure 1). For example, mice with a 2.5 mg corticosterone pellet had significantly fewer total thymocytes and significantly fewer cells in all but one of the major subpopulations than the naive group, whereas such decreases were only observed in the group with a 5 mg pellet after 24 hours (Figure 1). Similarly, the changes in subpopulation percentages were more pronounced after 72 hours (Figure 4) than after 24 hours (Figure 2). Some of the increases in cell number in adrenalectomized naive mice (ADX-N) or adrenalectomized mice with a placebo pellet implanted (ADX-P) compared to naive control that were observed in Figure 1 were not significant in the 72 hour experiment (Figure 3). It should be noted that these groups were essentially equivalent in all experiments, because adrenalectomy occurred 3 weeks before analysis in all mice. Evaluation of pooled, normalized data from 4 independent experiments indicates that compared to naive mice (100 ± 4.3%), ADX-N (143 ± 5.2%) and ADX-P (144 ± 8.3%) groups had significantly more total thymocytes. The mean number of nucleated cells per thymus in the naive (non-adrenalectomized) groups was 8.1 × 10^7 ^in these 4 experiments.

### Serum corticosterone concentrations indicate that corticosterone replacement with pellets yields appropriate corticosterone concentrations and that sub-physiological corticosterone concentrations (in ADX mice) are not sufficient to maintain thymus homeostasis

**Figure 5:**
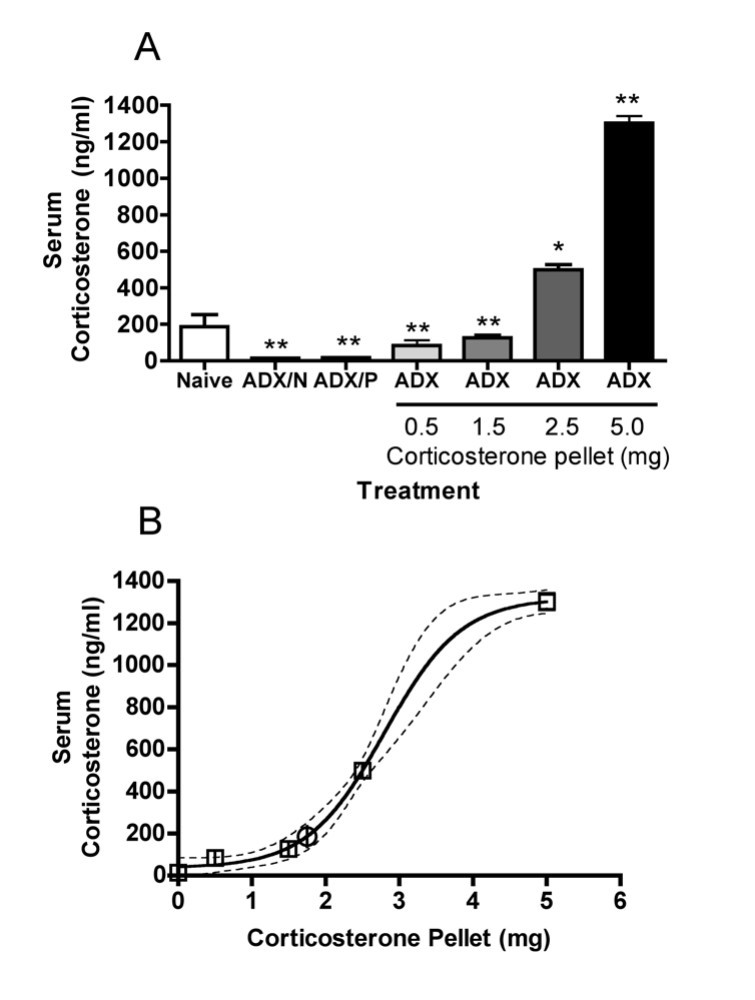
**Serum corticosterone concentration 24 hours after implantation of corticosterone pellets **Mice from one of the experiments noted in Figures 1 and 2 were bled prior to removal of the thymus, and serum corticosterone concentrations were determined by radioimmunoassay. In panel A, values shown are means ± SE (n = 5 mice/group), and values significantly different from the naive control are indicated by * (*p *< 0.05) or ** (*p *< 0.01). The treatments are described in the legend for Figure 1. In panel B, non-linear regression was used to determine a best-fit line through points from 0 (ADX-N) to 5 mg corticosterone pellets (open squares). The extrapolated pellet size required to produce the same corticosterone concentration shown for Naive mice in panel A is indicated by an open circle. Dotted lines indicate the 95% confidence interval for the regression line. The R-squared value for this relationship was 0.987.

The serum corticosterone concentration measured in naive mice was 185 ng/ml (Figure 5A). Normal corticosterone values in female B6C3F1 mice vary from less than 100 ng/ml to approximately 300 ng/ml in a circadian pattern [20].

Adrenalectomized mice had barely detectable concentrations of corticosterone in the serum, and corticosterone pellets containing increasing amounts of corticosterone produced increasing corticosterone concentrations in the serum. These results were obtained 24 hours after implantation of pellets. These serum corticosterone concentrations range from values that can be found in normal (unstressed) mice at certain times of the day [20] (observed in groups treated with 0.5 and 1.5 mg pellets) to values that are comparable to those measured in mice exposed to moderately or highly stressful conditions [20,21] (observed in mice treated with 2.5 or 5.0 mg pellets). Non-linear curve fitting was used to extrapolate the size of pellet that would be required to produce the same corticosterone concentration measured in naive mice. The extrapolated value (1.75 mg) is shown in Figure 5B. Thus, the effects of adrenalectomy on the thymus should not quite be reversed by a 1.5 mg pellet and should be more than reversed by a 2.5 mg pellet. The results shown in Figure 3 are reasonably consistent with this expectation. It seems appropriate to examine the 72-hour data in this regard (Figure 3), because this exposure period most likely represents the time required to achieve the maximum effects of corticosterone on the thymus. The slight differences from expected effects for some subpopulations may be related to the fact that pellets do not reproduce the circadian changes in corticosterone that occur in normal animals. These changes likely contribute to thymus homeostasis, and restoring corticosterone to a constant concentration may not mimic this effect precisely. Nevertheless, these results clearly demonstrate that corticosterone alone is able to reverse the effects of adrenalectomy and that this reversal occurs at concentrations within physiological levels. This suggests that corticosterone is the only adrenal product required to regulate thymocyte number. More importantly, these results conclusively demonstrate that corticosterone produced in the thymus is not sufficient to maintain normal thymocyte numbers.

### Differential sensitivity of thymic subpopulations as indicated by linear regression analysis

**Figure 6:**
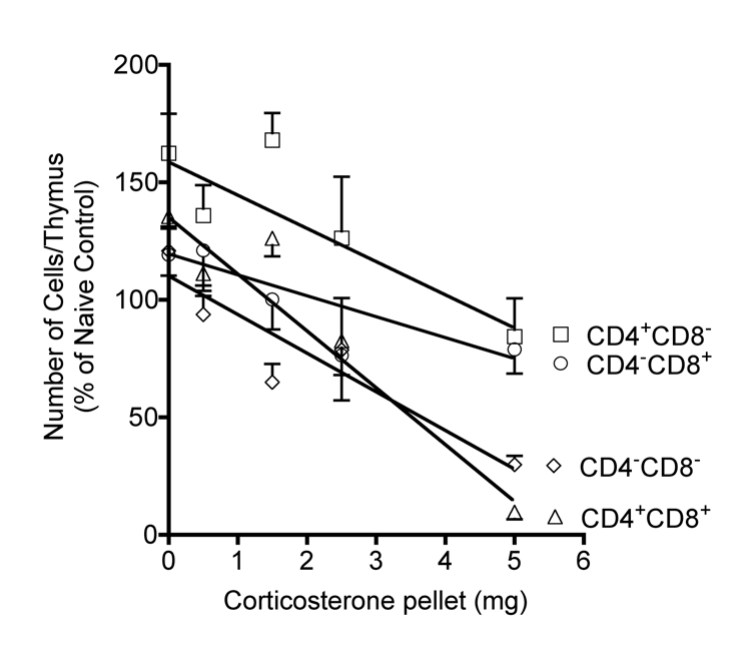
**Linear regression analysis of corticosterone pellet size (mg) and the number of cells per thymus for each of the 4 major subpopulations **The thymus was evaluated 24 hours after pellet implantation (~3–4 weeks after ADX), and all groups shown were ADX. Group size was 5–12. Values shown are mean ± S.E. obtained by normalizing groups to the mean value for the Naive group (defined as 100%). Results shown are pooled from two independent experiments, and statistically significant differences in the slope and intercept of each pair-wise combination are described in the text.

It is clear that thymic subpopulations differ in their sensitivity to high (stress-inducible) corticosterone concentrations. However, linear regression analysis (Figure 6) indicates that the sensitivities of the various cell populations are not as different as might be expected on the basis of the decreased percentage of CD4^+^CD8^+ ^cells and the increased percentages of the other subpopulations. The results demonstrate that increasing concentrations of corticosterone affect CD4^-^CD8^- ^and CD4^+^CD8^+ ^similarly, but the slope for CD4^+^CD8^+ ^cells is significantly greater (by the method of Zar as implemented by Prism software) than for CD4^-^CD8^- ^cells, indicating slightly greater sensitivity to high concentrations of corticosterone. The effect of increasing concentrations of corticosterone on CD4^+^CD8^- ^cells was significantly less than the effect on CD4^+^CD8^+ ^cells with regard to the slopes of the respective lines. The slope for CD4^-^CD8^+ ^cells was less than the slope for all other subpopulations, suggesting a lower sensitivity of these cells to corticosterone across the whole range of concentrations. However, the decrease for CD4^+^CD8^- ^cells and CD4^-^CD8^+ ^cells relative to naive control was essentially the same in mice treated with a 5 mg pellet, suggesting that the difference in sensitivity is minimal as the corticosterone concentration increases. Although non-linear models would likely have given better correlation coefficients than linear ones for some of these data, linear models facilitate comparison. In addition, the Runs Test was conducted for all linear regression analyses, and the non-linear component was not significant for any of these data.

### Role of changes in the rate of apoptosis in decreased and increased cell number in the thymus of ADX mice with or without a corticosterone pellet

**Figure 7:**
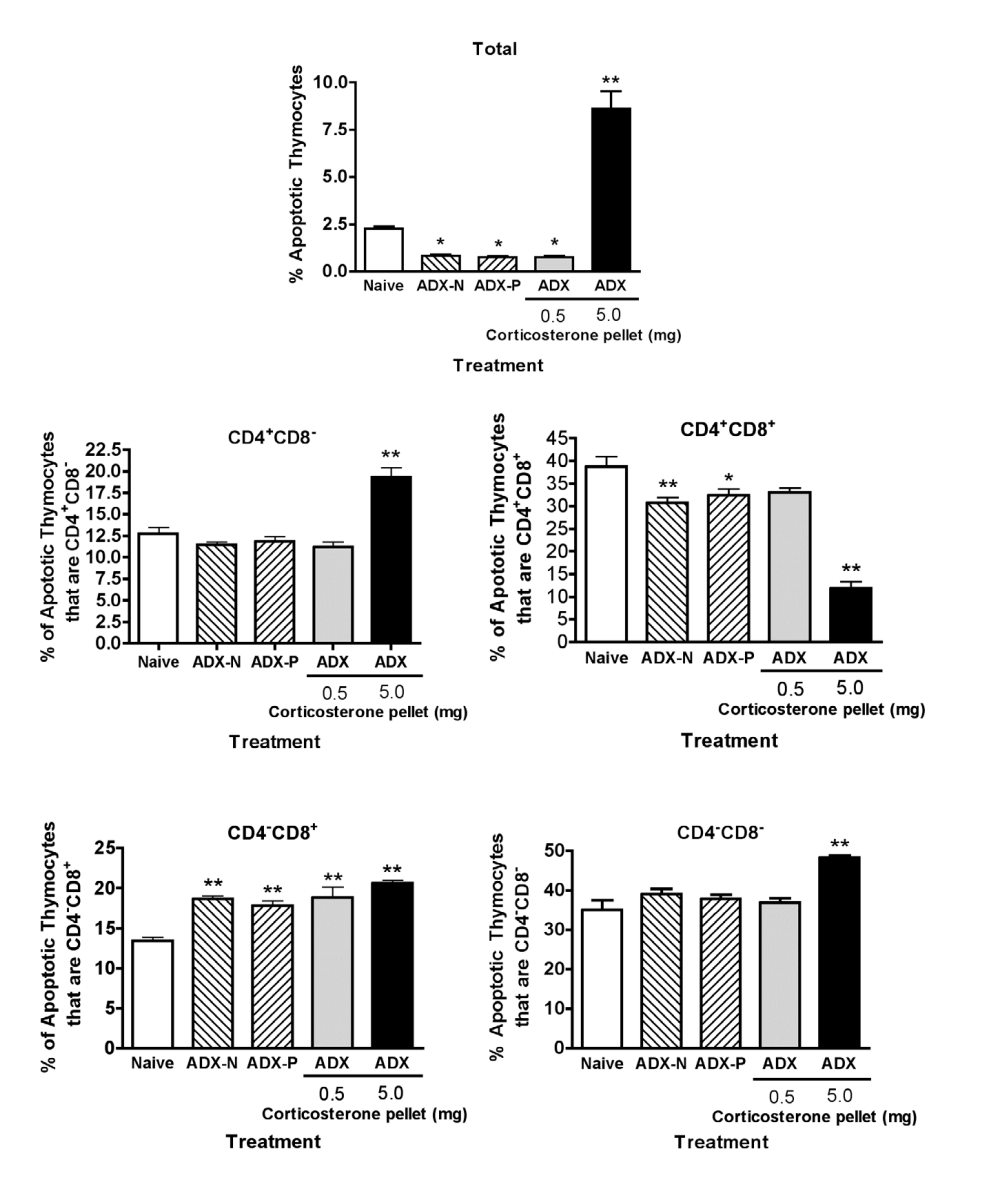
**Apoptosis in the thymus in response to sub-physiological and stress-inducible concentrations of corticosterone **Apoptosis was evaluated using the TUNEL technique with fluorescein-labelled dUTP. The cells were also surface labelled with fluorescent-labelled antibodies to CD4 and CD8. Values shown represent means ± SE for groups of 5 mice. The values in the upper panel represent the percentage of apoptotic cells in the thymus. The other values represent the percentage of apoptotic cells that are within each of the major 4 subpopulations in the thymus. Groups significantly different from the naive group (by ANOVA followed by Dunnett's test) are shown by * (*p *< 0.05) or ** (*p *< 0.01).

There are several mechanisms by which corticosterone might act to alter the number of cells in each subpopulation in the thymus. Although induction of apoptosis is generally regarded to be the major mechanism [1-3,22], altering differentiation or proliferation of thymocytes, altering the development of pro-thymocytes in the bone marrow, or altering pro-thymocyte or thymocyte trafficking to or from the thymus are all possible mechanisms. To determine if changes in apoptosis may play a role in the effects noted in this study, apoptosis was evaluated using two criteria: cell size (indicated by forward scatter) and TUNEL labelling for DNA fragmentation. As shown in Figure 7, the results demonstrate that the percentage of apoptotic cells in the thymus was decreased in mice that had sub-physiological concentrations of corticosterone in the serum (ADX-naive, ADX-placebo, and ADX mice with a 0.5 mg corticosterone pellet). The percentage of apoptotic cells was substantially increased in mice with a high (stress-inducible) concentration of corticosterone in the serum (caused by a 5 mg pellet).

Initially, changes in the percentage of apoptotic cells in the various subpopulations did not seem entirely consistent with the changes in the percentages of each subpopulation in the thymus (compare Figure 2 and Figure 7). Three-color flow cytometry allowed determination of the percentage of apoptotic cells in each subpopulation. In naive mice, most apoptotic cells were CD4^+^CD8^+^, as expected, with a substantial percentage in the CD4^-^CD8^- ^category and lesser percentages of the mature single positive categories. The percentages of apoptosis in all populations increased in mice treated with a 5.0 mg corticosterone pellet, except CD4^+^CD8^+ ^cells, for which the percentage decreased. This almost certainly reflects the fact that this subpopulation was depleted by 24 hours of elevated corticosterone concentrations (Figure 1), and at least some of the remaining CD4^+^CD8^+ ^cells were likely glucocorticoid resistant [23]. However, it should also be noted that an increase in apoptosis was only observed in mice treated with a 5 mg pellet, not in mice treated with a 0.5 mg pellet. In contrast, a 0.5 mg pellet diminished the significant increase in cell numbers caused by adrenalectomy (Figure 3). This suggests the possibility that mechanisms other than apoptosis may also be involved glucocorticoid-mediated homeostasis in the thymus. The large percentage of CD4^-^CD8^- ^cells in the apoptotic population was not entirely unexpected, because there is a developmental checkpoint that can lead to death in CD4^-^CD8^- ^cells that do not productively rearrange a TCR β chain [24]. In the human thymus, the percentage of apoptotic CD4^-^CD8^- ^is lower than we noted (13% in humans vs. 34% in the present study) [25]. However, the human thymuses in that study were obtained from newborns, and the differences could thus reflect age as well as species differences.

### Effect of ADX with or without corticosterone pellets on immature single positive cells in the thymus

**Figure 8:**
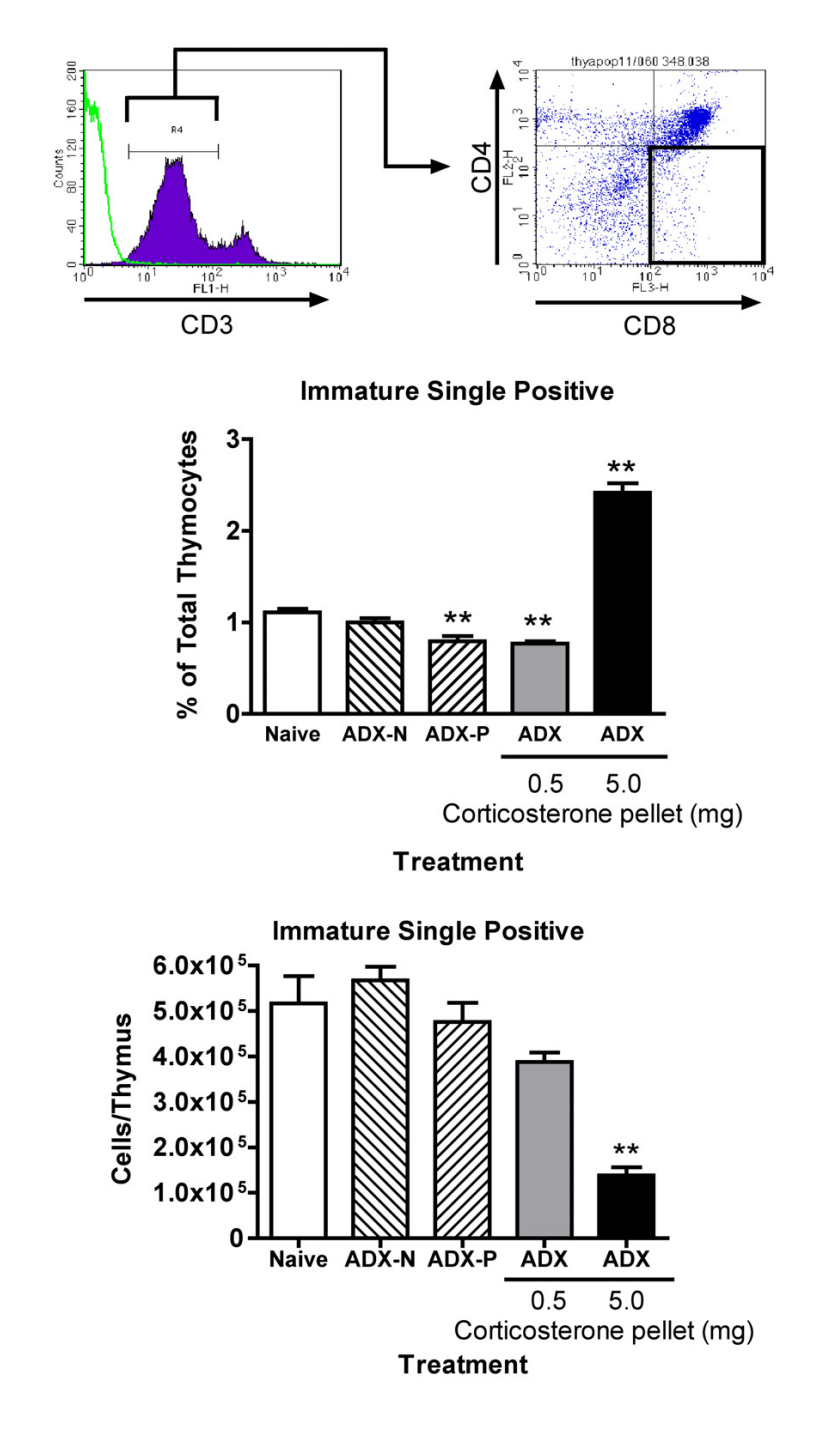
**Effect of sub-physiological and stress-inducible concentrations of corticosterone on immature single positive (CD8^+^) thymocytes **The histograms at the top of this figure illustrate the region containing cells that express low levels of CD3 (the larger purple peak on the histogram to the left, in which the isotope control is indicated by a green line), and the subsequent analysis of those cells for CD4 and CD8. The cells in the lower right quadrant (outlined with bold lines) are CD3^low^CD8^+^, immature single positive cells. The values shown in the graphs are means ± SE for groups of 5 mice representing either the percentage of immature single positive cells in the thymus or the total number of these cells in the thymus (obtained by multiplying the percentage by the total thymus cell number for each mouse). Groups significantly different from the naive group (by ANOVA followed by Dunnett's test) are shown by ** (*p *< 0.01).

Single positive CD8 cells that express low levels of CD3 (and TCR) are apparently the immediate precursors for CD4^+^CD8^+ ^cells in the thymus [24]. One report indicates that these cells are more susceptible than mature single positive cells to glucocorticoids [19]. Results shown in Figure 8 are consistent, at least in part, with this finding. The increase in the percentage of this subpopulation in mice treated with a 5 mg pellet (2.4 fold) was substantially less than the increase in mature single positive cells (~5-fold) (Figure 2). In addition, the number of immature CD8^+ ^cells was decreased to a greater extent than for mature single positive cells at this time point (Figure 1). This suggests that immature single positive cells are more sensitive to high levels of corticosterone than mature single positive cells, though they are apparently less sensitive than CD4^+^CD8^+ ^and CD4^-^CD8^- ^cells. The number of immature single positive cells did not increase significantly in ADX mice that did not receive a corticosterone pellet, indicating that homeostasis of this cell type is not affected by physiological levels of corticosterone. This further illustrates that it is not appropriate to infer the effects of physiological concentrations of glucocorticoids on various cells types on the basis of the action of pharmacological concentrations of glucocorticoids. The percentage of immature single positive cells in the thymus of untreated mice in our study was comparable to values noted by others [19,26].

### Relationship of present results and results from other studies

The findings reported here are consistent with some, but not all, results from other laboratories. The study most relevant to the present one involved the use of transgenic mice that express glucocorticoid receptor at higher than normal or lower than normal levels [4]. Comparing changes in the number of thymocytes in various subpopulations in mice expressing twice the normal level of glucocorticoid receptor in the thymus with our results at moderately elevated corticosterone concentrations (2.5 mg pellet, figure 3) indicates some similarities and some differences. In both studies, the total number of thymocytes was decreased significantly. However, expression of higher levels of glucocorticoid receptor caused significant suppression of cell numbers for CD4^+^CD8^+^, CD4^-^CD8^-^, and CD4^+^CD8^- ^cells, but not CD4^-^CD8^+ ^cells [4]. In contrast, the 2.5 mg pellet in adrenalectomized mice (a situation that should be analogous to higher levels of glucocorticoid receptors with normal corticosterone concentrations) caused significant decreases in cell number in CD4^+^CD8^+^, CD4^-^CD8^+^, and CD4^-^CD8^- ^cells, but not in CD4^+^CD8^- ^cells (Figure 3). In mice expressing lower than normal levels of glucocorticoid receptor in the thymus (due to incorporation of anti-sense DNA under the control of the lck promoter) [4], the pattern of change was very similar to that which we observed in adrenalectomized mice (with no corticosterone pellet). In both cases, the significant increases in cell number were noted only for the CD4^+^CD8^- ^and CD4^+^CD8^+ ^subpopulations. Cell number in the other two major subpopulations was increased slightly, but not significantly.

The overall relationships between these results might be explained by a report indicating that normal expression of glucocorticoid receptor in the thymus is a very dynamic process, with substantial changes in expression in different subpopulations of cells [19]. In addition, the evidence suggests that sensitivity of the various subpopulations to glucocorticoids is not always strictly dependent on the amount of glucocorticoid receptor expressed. Thus, other factors that change during the development of T cells play an important role in sensitivity to glucocorticoids. Causing excess production of glucocorticoid receptor in all cells of the thymus (as in transgenic mice with an extra glucocorticoid receptor gene, transcribed in all thymocytes under the control of the lck promoter) [4] would not be likely to produce the same differences in glucocorticoid receptor levels among cellular subpopulations in the thymus as noted in normal animals (in which glucocorticoid receptor levels vary in different subpopulations). A portion of the glucocorticoid receptor production would be subject to the normal dynamic regulatory process, but a portion of production (the portion under the control of the lck promoter) would not. This may explain the differences in the results obtained using transgenic mice with elevated levels of glucocorticoid receptor [4] as compared to our results using elevated corticosterone concentrations. However, expression of glucocorticoid receptor anti-sense RNA in the thymus in a uniform manner [4] seems to have produced similar results as decreased corticosterone concentrations (in adrenalectomized animals) (Figs. 1,2,3,4,). This may reflect the fact that the action of anti-sense RNA in a particular cell type would be expected to be proportional to the amount of glucocorticoid receptor expressed in that cell. Thus, the normal differences between subpopulations with regard to glucocorticoid sensitivity might be retained. Thus, it is not surprising that the results with anti-sense glucocorticoid receptor transgenic mice are comparable to those for adrenalectomized mice in our study with regard to the differential increase in cell number for different subpopulations. It is not clear why no increase in thymus cellularity or changes in subpopulations were noted by other investigators using a conditional knockout system to produce mice in which the thymus contains little glucocorticoid receptor [14]. However, glucocorticoid receptor knockout mice can express portions of the glucocorticoid receptor, which may have unexpected functions [27]. Such contradictory findings with transgenic approaches have been common in this field of research (see Introduction), indicating a useful role for classical pharmacological approaches such as those in the present study.

The relationship between the results reported here and results from studies on the interactions between glucocorticoids and self-antigen in positive selection is not clear. A recent study indicates that activation through the TCR down regulates SRG3, a protein that associates with the glucocorticoid receptor and increases sensitivity to glucocorticoids [28]. This has been proposed as an explanation for the decreased sensitivity of mature single positive thymocytes as compared to non-mature double positive thymocytes to high concentrations of glucocorticoids. However, as already noted, this pattern did not seem to apply when comparing the effects of sub-physiological and physiological concentrations of glucocorticoids, i.e., the CD4^+^CD8^- ^cells increased to a greater extent than CD4^+^CD8^+ ^cells in mice with sub-physiological corticosterone concentrations. This suggests that the observed changes in SRG3 may not account for differences in sensitivity of cells in different subpopulations to physiological concentrations of corticosterone. This leaves open the question of what does mediate those differences and the role (if any) of TCR signalling. It would be of interest to explore this with TCR transgenic mice.

One study in which adrenalectomy has been used to evaluate the effects of glucocorticoids on cellular subpopulations in the thymus yielded different results than those reported here. In that study, there was no increase in total cell number in the thymus, and there were no changes in subpopulation percentages in adrenalectomized mice two weeks after adrenalectomy [18]. The basis for the difference in this result and the results of other studies, which indicate increased numbers of thymocytes in adrenalectomized mice or rats [15-17] is not clear. In a study in which one of the authors of the present report (E. L. P.) was involved, there was a greater increase in cell number in the thymus in adrenalectomized mice than in the present study [16]. Perhaps because the overall increase in cell number was greater, the increases in all subpopulations were significant [16]. The age and housing conditions (and resulting environmental stress levels) of the control group probably plays an important role in this regard, and it would be very difficult to assure precisely the same conditions for the control group in every case. Nevertheless, the results reported here along with results from most other adrenalectomy studies and results from one study using transgenic mice [4] indicate that physiological (non-stress) concentrations of corticosterone normally decrease the number of cells in the thymus.

## Conclusions

The results presented here do not directly demonstrate the extent to which corticosterone contributes to death by neglect or the extent to which it contributes to the death of negatively selected thymocytes. The fact that both CD4^+^CD8^+ ^and CD4^+^CD8^- ^cells are increased in number in ADX mice is consistent with the idea that death by neglect of CD4^+^CD8^+ ^cells is mediated by corticosterone. The increase in CD4^+^CD8^- ^cells could be explained by the failure of CD4^+^CD8^+ ^cells to die before reaching maturity, as they would have done in the presence of corticosterone. The observation that CD4^-^CD8^+ ^cells are not increased to the same extent as CD4^+^CD8^- ^cells is exactly what would have been predicted if sub-physiological concentrations of corticosterone allow survival of cells that would normally die by neglect. Whereas maturation of CD4^+^CD8^+ ^cells to CD4^-^CD8^+ ^cells requires MCH class I-dependent signals, maturation to CD4^+^CD8^- ^status can be MHC-independent and apparently occurs by default [29]. Thus, cells that are not selected positively may preferentially mature to CD4^+^CD8^- ^cells before dying by corticosterone-mediated apoptosis. However, sub-physiological concentrations of corticosterone apparently allow these non-selected cells to survive.

## Methods

### Animals and animal care

Female C57BL/6 × C3H F1 (B6C3F1) mice were used in this study. Normal, adrenalectomized (ADX), and sham adrenalectomized mice were purchased from Charles River Labs (Wilmington, MA). The mice were allowed to recover from shipping stress for at least two weeks before use in experiments, and surgery was performed approximately one week before shipping. Thus, mice were evaluated at least three weeks after surgery at an age of 8–12 weeks. Mice were maintained on a 12 hour light/dark cycle, with free access to lab chow and water, except that ADX mice were given water with 0.9% sodium chloride. Sentinel mice housed periodically in the same room as the mice used in this study were negative for common adventitious agents and pathogens of mice. Animal care and use was in accord with the regulation of LSU Health Sciences Center and the NIH Guide for Care and Use of Laboratory Animals. The animal facility in which the mice were maintained is approved by the American Association for Accreditation of Laboratory Animal Care.

### Implantation of timed release corticosterone pellets

Timed-release corticosterone pellets and placebo pellets were purchased from Innovative Research of America (Sarasota, FL). The pellets are designed to yield constant blood levels of corticosterone for 3 weeks. Pellets were implanted subcutaneously in the scapular area of mice that were anesthetized with sodium pentobarbital (55 mg/kg) and inhalation of methoxyflurane, as described in a previous study [2]. The incision was closed with a surgical staple. The entire process was conducted aseptically. Mice were allowed to recover on a heating pad prior to being returned to their home cages. Parameters were evaluated after 24 hours in two experiments and after 72 hours in a third experiment.

### Preparation of cells and flow cytometry

In two experiments, mice were euthanized by CO_2 _inhalation and the thymus was removed for analysis. In one experiment mice were euthanized by decapitation, trunk blood was obtained and allowed to clot, and serum was isolated after centrifugation. The serum was used to determine corticosterone concentration, using a radioimmunoassay kit (Diagnostic Products Corporation, Los Angeles, CA) as described previously [30,31]. The thymus was then removed from each mouse. Single cell suspensions were prepared in 3 ml of RPMI 1640 by pressing the organs between the frosted ends of sterile glass microscope slides, as in previous studies [1,2]. After centrifugation, the cells were resuspended in 3 ml of RPMI 1640, 20 μl samples were taken, diluted in 10 ml of Isoton II isotonic buffered saline (Coulter Corp., Miami, FL), and counted using an electronic cell counter (Coulter Model Z1). Cells were adjusted to 2 × 10^7 ^per ml, and 50 μl was added to the wells of a 96-well V-bottom microplate. Antibodies diluted in 50 μl of FACS buffer (phosphate buffered saline without calcium and magnesium plus 0.1% bovine serum albumin and 0.1% sodium azide) were added to appropriate wells. In each experiment in which multiple antibodies were used, controls included cells labeled with each antibody singly, cells labeled with each isotype control antibody singly, cells labeled with all isotype control antibodies together, and unlabeled cells. The following antibodies were used: anti-CD4 (GK1.5) labeled with phycoerythrin (PE), anti-CD8a labeled with Cychrome, and anti-CD3 labeled with fluorescein isothiocyanate (FITC). These antibodies and matching isotype controls were obtained from BD Pharmingen. Titration of the antibodies indicated that a 1:8 dilution of anti-CD4 and anti-CD8 and a 1:5 dilution of anti-CD3 were appropriate for this study. After labeling for 30 min at 4°C, the cells were washed, fixed with 1% paraformaldehyde (EM Sciences, Ft. Washington, PA), washed again, and resuspended in FACS buffer. Samples were diluted in Isoton II (0.4 ml) for analysis. Cells were analyzed using a FACScan flow cytometer (Becton-Dickinson). A gate was set using forward scatter and side scatter to exclude debris, erythrocytes, and clumps of cells. All cells within this gate were then analyzed for CD3, CD4, and CD8.

In some experiments, cells were labeled to detect DNA fragmentation instead of CD3. Cells were first labeled with anti-CD4 (phycoerythrin) and anti-CD8 (cychrome) as described above, then the cells were fixed with 4% paraformaldehyde (in phosphate buffered saline). A terminal dUTP nick end labeling (TUNEL) kit from Boehringer-Mannheim (Indianapolis, IN) with fluorescein-labeled dUTP was used to label apoptotic cells. Flow cytometry was used to identify apoptotic cells by two criteria. Cells that were small (as indicated by forward scatter) and labeled with fluorescein (indicating DNA fragmentation) were regarded to be apoptotic.

### Statistical analysis

Values significantly different from the naive (untreated) control group were determined by analysis of variance (ANOVA) followed by Dunnett's post hoc test. Statistical analysis, linear regression, and non-linear regression analysis were performed using Prism 4.0 software (GraphPad, Inc., San Diego, CA). Comparison of slope or intercept of pairs of regression lines was done using the method of Zar [32] as implemented by Prism software.

## Authors' contributions

The authors contributed approximately equally to conceiving, designing, and conducting these experiments. S.B.P. wrote the manuscript, and E.L.P. revised it.
